# Why should being visible on the road? A challenge to prevent road traffic injuries among pedestrians in Iran

**DOI:** 10.5249/jivr.v7i2.490

**Published:** 2015-07

**Authors:** Davoud Khorasani Zavareh, Katarina Bohm, Hamidreza Khankeh, Mohammad Taghi Talebian, Reza Mohammadi, Maryam Bigdeli, Maaret Castren

**Affiliations:** ^*a*^Road Traffic Injury Research Center, Tabriz University of Medical Sciences, Tabriz, Iran.; ^*b*^Department of Clinical Science and Education, Södersjukhuset, Karolinska Institutet, Stockholm, Sweden.; ^*c*^Department of Health in Emergency and Disaster, University of Social Welfare and Rehabilitation, Tehran, Iran; ^*d*^Department of Emergency Medicine, School of Medicine, Tehran University of Medical Sciences, Tehran, Iran.; ^*e*^Department of Social Medicine, Karolinska Institutet, Stockholm, Sweden.; ^*f*^Social Determinants of Health Research Center, Urmia University of Medical Sciences, Urmia, Iran.

As a major public health problem, road traffic injuries (RTIs) affect all social groups of the society.^1^ Every year, around 1.3 million people die and 20-50 million people get injured or disabled as a result of the RTIs worldwide.^2^ The figure of RTIs is more alarming in low and middle-income countries (LMICs).^1-2^ RTIs in Iran are very important public health problems in the whole country. ^3-6^ When World Health Organization launched its call to action, it invited members of the public to be a part of the solution.^1^ The initiative focused on five important courses of action for the general public of which included: no speeding; wearing a seat-belt; wearing a helmet; never drink and drive; and being visible on the road. Being seen on the road is a fundamental requirement for safety of all road users, particularly vulnerable road users.^2^ Inadequate visibility plays a key role in RTIs during both day and night for pedestrians, cyclists and motorcyclists as well as car drivers.^1^ Being visible on the road seems to be a neglected action and hence it is still a major challenge, particularly in low and middle- income countries like Iran. Accordingly, it was decided to explore barriers related to pedestrians’ visibility on the road and to find suggestions for improvement based on road users’ experiences in Iran.

Seventeen face-to-face interviews were conducted with road users including: pedestrian, motorcyclist, cyclist and car drivers between February-June 2013. A qualitative approach by means of content analysis was used to analyze the data. Findings indicated that barriers related to pedestrians’ visibility included a lack of knowledge of the importance of being seen, negative attitude towards using reflectors, a sense of being unusual when using reflectors, the significance of dark clothes as well as cultural support on wearing dark clothes and a lack of production of reflectors in the country. Suggestions for improvement included a public education campaign, effective law legislation, more rigorous law enforcements in terms of being visible on the road, more facilities for production of reflective materials and a more importantly further availability of reflectors in the country.

In sum, the major barriers in terms of being visible of pedestrians on the road were identified as a lack of knowledge of importance of visibility and negative attitude in using reflective materials in both public and stakeholders. Suggestion for improvement of being visible on the roads covered educational campaigns and stricter law enforcements as well as more availability of reflectors in the society. 
